# Nitrogen-fixing Ability of a Thermophilic Sulfate-reducing Bacterium in the Genus *Thermodesulfovibrio* Isolated from a Terrestrial Hot Spring in Japan

**DOI:** 10.1264/jsme2.ME25030

**Published:** 2025-09-18

**Authors:** Toko Hisano, Arisa Nishihara, Shin Haruta

**Affiliations:** 1 Department of Biological Sciences, Tokyo Metropolitan University, 1–1 Minami-Osawa, Hachioji, Tokyo 192–0397, Japan; 2 Department of Biological Sciences, Purdue University, West Lafayette, IN 47907, USA; 3 Bioproduction Research Institute, The National Institute of Advanced Industrial Science and Technology (AIST), 1–1–1 Higashi, Tsukuba, Ibaraki 305–8566, Japan

**Keywords:** N_2_ fixation, thermophile, sulfate-reducing bacteria

## Abstract

Nitrogen-fixing sulfate-reducing bacteria have not yet been exami­ned in thermal environments. In this study, strain TK110, belonging to the genus *Thermodesulfovibrio*, was successfully isolated from a geothermal spring using an NH_3_-free inorganic medium. Strain TK110 harbored genes associated with the Calvin–Benson–Bassham cycle and nitrogen fixation-related genes, *nifHDKENXIIB*. Nitrogenase activity was assessed using an acetylene reduction assay and detected in strain TK110 under autotrophic conditions, as well as in *Thermodesulfovibrio yellowstonii* DSM 11347^T^ under heterotrophic conditions at 65°C. To the best of our knowledge, this is the first study to demonstrate nitrogen fixation by thermophilic sulfate-reducing bacteria.

Nitrogen (N_2_) fixation has been reported in 16 phyla in *Bacteria* and 1 phylum in *Archaea* (Mus *et al.*, 2019), with extensive studies on the ecology and evolution of N_2_-fixing microorganisms (Mehta *et al.*, 2003; Hamilton *et al.*, 2011; Zehr, 2011; Pajares and Bohannan, 2016). However, the diversity of thermophilic N_2_-fixing microorganisms growing at temperatures >60°C remains unclear, as only a limited number of isolates have been reported to date. These include a methanogen (*Methanocaldococcus* sp. in the phylum *Methanobacteriota* at 92°C) (Mehta and Baross, 2006), a fermenter (*Caldicellulosiruptor* sp. in the phylum *Bacillota* at 78°C) (Chen *et al.*, 2021), an aerobic chemolithotroph (*Hydrogenobacter* sp. in the phylum *Aquificota* at 70°C) (Nishihara *et al.*, 2018b), and an oxygenic phototroph (*Synechococcus* sp. in the phylum *Cyanobacteriota* at 60°C) (Steunou *et al.*, 2006). Dissimilatory sulfate reduction is distributed across diverse phylogenetic groups, with sulfate-reducing bacteria and archaea inhabiting various environments, including geothermal springs (Mori *et al.*, 2003; Qian *et al.*, 2019). However, the highest reported temperature for N_2_ fixation by sulfate-reducing bacteria or archaea is 37°C (Sayavedra *et al.*, 2021).

In our previous study, N_2_-fixing activity via dissimilatory sulfate reduction was indicated through *ex situ* incubation experiments using microbial mats from a geothermal spring (Nishihara *et al.*, 2018a). Microbial mats collected at Nakabusa Hot Springs, Nagano, Japan, exhibited acetylene reduction activity at 70°C under anaerobic conditions. This acetylene reduction activity was inhibited by molybdate, a known inhibitor of sulfate reduction (Nishihara *et al.*, 2018a). Additionally, the acetylene reduction activity of the mats was enhanced by the addition of H_2_ and CO_2_, suggesting the existence of thermophilic, N_2_-fixing chemo­lithotrophic sulfate-reducing bacteria that had not yet been cultivated.

In the present study, we isolated a thermophilic bacterium from Nakabusa Hot Springs using a sulfate-containing, nitrogen compound-free inorganic medium under an N_2_:CO_2_:H_2_ atmosphere. We characterized the mole­cular phylogeny, genetic contents, and N_2_-fixing activity of the bacterial isolate.

Microbial mats were collected from hot spring water at 54°C in Nakabusa Hot Springs (36°23′20″N, 137°44′52″E, Nagano, Japan) in July 2022. Pieces of the microbial mats‍ ‍were inoculated into a modified JCM479 medium (https://jcm.brc.riken.jp/) and cultivated at 65°C under an N_2_:CO_2_:H_2_ atmosphere. The modified JCM479 medium was prepared by removing organic compounds (Na-lactate, Na-pyruvate, and yeast extract) from the original recipe. The medium contained (L^–1^): 2.78‍ ‍g Na_2_SO_4_, 0.54‍ ‍g NH_4_Cl, 0.15g KH_2_PO_4_, 0.22‍ ‍g MgCl_2_·6H_2_O, 0.17‍ ‍g CaCl_2_·2H_2_O, and 2.67‍ ‍g NaHCO_3_, supplemented with trace elements, vitamins, cysteine-HCl, and Na_2_S. pH was adjusted to 6.5. To inhibit methanogens, 20‍ ‍mmol L^–1^ of 2-bromo-ethane sulfonate (BES) was added (Gunsalus *et al.*, 1978). Twenty milliliters of the medium was placed into a 70-mL glass vial, sealed with a butyl rubber stopper and aluminum cap, and flushed with N_2_:CO_2_ (4:1 [v:v]). The medium was autoclaved after pressurization with H_2_:CO_2_ (4:1 [v:v]) gas to approximately 0.2 MPa (*i.e.*, N_2_:CO_2_:H_2_=2:1:2 [v:v:v]). Cultures were subcultured in fresh medium every 7–10 days. After more than 15 subcultivations, the culture was inoculated into the nitrogen compound-free medium that was prepared by removing NH_4_Cl from the modified JCM479 medium (NH_3_-free modified JCM479 medium) and‍ ‍subcultivated. Following seven subcultivations in this medium, single colonies were isolated by the roll-tube method using the NH_3_-free modified JCM479 medium containing 1.0% (w/v) gellan gum, solidified in a 70-mL glass vial under an N_2_:CO_2_:H_2_ atmosphere. After cultivation at 65°C, colonies were picked up and reinoculated into the fresh medium. This single colony isolation process was repeated twice, followed by three rounds of dilution-to-extinction cultivation. The purity of the isolates was confirmed via microscopic observations (Axio Imager 2; Carl Zeiss) and 16S rRNA gene sequencing (see below). Isolates were maintained in the NH_3_-free modified JCM479 medium with or without Na-acetate (2‍ ‍mmol L^–1^) and without BES.

Bacterial cells were collected via centrifugation after cultivation in the NH_3_-free modified JCM479 medium, and DNA was extracted using the Template Prepper for DNA (NIPPON GENE). The 16S rRNA gene fragment was PCR-amplified using the 27F and 1492R primers (Lane, 1991; Suzuki and Giovannoni, 1996) and sequenced using the BigDye terminator kit v3.1 on an ABI3130 Genetic Analyzer (Applied Biosystems) as previously described (Hirose *et al.*, 2016). Sequences were compared with the DDBJ/EMBL/GenBank databases using BLAST (Altschul *et al.*, 1997).

In the genomic ana­lysis, a bacterial isolate was cultivated in 150‍ ‍mL of the modified JCM479 medium containing acetate for 12 days to collect cells. Bacterial DNA was extracted using the Qiagen Genomic-tip 100/G (Qiagen) and‍ ‍sequenced using the Revio (PacBio) platform by Bioengineering Lab. A sequencing library was prepared using the SMRTbell Express Template Preparation Kit 3.0 and the SMRTbell gDNA Sample Amplification Kit (PacBio). The library was sequenced with the Revio (PacBio) using the Revio Polymerase Kit (PacBio). A total of 30,863 high-fidelity reads with an average length of 7,938 bp were obtained using SMRT Link ver. 13.0.0.207600 (PacBio). Reads were trimmed using lima (ver. 2.9.0) and duplicate PCR reads were removed using pbmarkdup (ver. 1.0.3). High-quality reads filtered using Filtlong ver. 0.2.1 (https://github.com/rrwick/Filtlong) to remove reads of <1,000 bases were assembled using Flye v2.9 (Kolmogorov *et al.*, 2019). The generated contigs were exami­ned using Bandage v0.8.1 (Wick *et al.*, 2015) and CheckM2 ver. 1.0.1 (Parks *et al.*, 2015; Chklovski *et al.*, 2023). Genome annotation was performed using the DFAST pipeline (Tanizawa *et al.*, 2018). Default parameters were used for all software ana­lyses.

Bacterial growth was monitored using glass culture tubes sealed with butyl rubber stoppers by measuring optical density (OD) at 660‍ ‍nm with a miniphoto 518R spectro­photometer (Taitec). Nitrogenase activity was assessed using the acetylene reduction assay (Dilworth, 1966). *Thermodesulfovibrio yellowstonii* DSM 11347^T^ (Henry *et al.*, 1994) and *Thermodesulfovibrio thiophilus* JCM 13216^T^ (Sekiguchi *et al.*, 2008) were obtained as reference strains from the German Collection of Microorganisms and Cell Cultures (DSMZ) and the Japan Collection of Microorganisms (JCM), respectively. *T. yellowstonii* and *T. thiophilus* were maintained in the modified JCM479 medium containing Na-lactate (2‍ ‍mmol L^–1^) at 65 and 55°C, respectively, under an N_2_:CO_2_:H_2_ atmosphere. Bacterial strains were precultured in the NH_3_-free modified JCM479 medium with or without Na-lactate. A 0.2-mL aliquot of the stationary-phase culture was inoculated into 20‍ ‍mL of fresh medium in 70-mL glass vials and cultivated under an N_2_:CO_2_:H_2_ atmosphere. At the growing phase, 0.5‍ ‍mL of the culture solution was collected from the vial and mixed with 0.5‍ ‍mL of 10% formalin neutral buffer solution (Fujifilm Wako Pure Chemical) to fix cells for cell counting. The vial’s gas phase was then replaced with Ar:CO_2_ (4:1 [v:v]) before injecting 5‍ ‍mL of 99.9999% acetylene gas and pressurizing H_2_:CO_2_ (4:1 [v:v]) gas to approximately 0.2 MPa. After an incubation at 65°C for 34 h, ethylene production in‍ ‍the gas phase was quantified using a GC-2014 gas chromatograph equipped with a flame ionization detector (Shimadzu) and 80/100 Porapak T column (GL Science) as previously described (Chen *et al.*, 2021). Cell counts were performed after staining with SYTO9 using a fluorescence cell counter (Countstar Mira FL Pro; Shanghai RuiYu Biotech) according to the manufacturer’s instructions.

Microbial mats collected from Nakabusa Hot Springs were cultivated at 65°C in the modified JCM479 medium under an N_2_:CO_2_:H_2_ atmosphere to obtain a stable enrichment culture. The culture was repetitively subcultured in the NH_3_-free modified JCM479 medium, and an isolate was obtained through single colony isolation and dilution-to-extinction. This isolate was designated as strain TK110. Colonies of strain TK110 in gellan gum-solidified medium comprised black-colored spheres (data not shown), and cells were curved rods of 2–3‍ ‍μm in length (Fig. S1). A BLAST search and mole­cular phylogenetic ana­lysis based on the 16S rRNA gene sequence indicated that strain TK110 belonged to the genus *Thermodesulfovibrio* in the phylum *Nitrospirota* (Fig. S2). The 16S rRNA gene sequence of strain TK110 (1,457 bp) exhibited 99.04, 98.63, and 97.87% identities to those of its close relatives, *Thermodesulfovibrio autotrophicus* 3907-1M, *Thermodesulfovibrio obliviosus* 3462-1, and *Thermodesulfovibrio aggregans* TGE-P1, respectively.

The whole genome of strain TK110 was sequenced, revealing a single chromosome with a length of 1,953,749 bp, 2,004 protein-coding genes, 3 rRNA operons, and 47 tRNA genes. Estimated completeness was 100% and contamination was 0.3%. Genes encoding the Calvin-Benson-Bassham cycle mediated by form III RubisCO were found‍ ‍(Table S1); however, none of the six other carbon fixation pathways were detected, as recently reported for *T.‍ ‍autotrophicus* 3907-1M (Frolov *et al.*, 2019; Maltseva *et‍ ‍al.*, 2024). Additionally, N_2_-fixation-related genes, *nifHDKENXIIB*, were identified in the genome of strain TK110 (Fig. 1). These genes were also present in some species in the genus *Thermodesulfovibrio*: *T. yellowstonii*, *T. islandicus*, *T. aggregans*, and *T. hydrogeniphilus*, but were absent in *T. autotrophicus*, *T. thiophilus*, and *T. obliviosus*. However, no previous reports have detected N_2_ fixation in any *Thermodesulfovibrio* species.

Strain TK110 was successfully cultivated in the NH_3_-free modified JCM479 medium under an N_2_:CO_2_:H_2_ atmosphere. Growth was confirmed through repeated subcultivation, with the OD of the culture in glass test tubes increasing from OD=0.014±0.007 at the inoculation to OD=0.053±0.008 at the stationary phase after 9 days of cultivation. Growth was not detected in the presence of 20‍ ‍mmol L^–1^ of sodium molybdenum oxide, an inhibitor of sulfate reduction (Peck, 1959). *T. yellowstonii* and *T. thiophilus* did not grow chemolithotrophically, and their N_2_-fixing growth was assessed in the presence of lactate. N_2_-fixing growth was observed in *T.‍ ‍yellowstonii*, with OD increasing from 0.016±0.010 to 0.041±0.005 over 9 days, whereas *T. thiophilus* showed no growth (OD=0.025±0.003 to 0.028±0.002).

Acetylene reduction assays were conducted using strain TK110 and *T. yellowstonii* cells in the growing phase in the NH_3_-free medium. Ethylene production was measured after a 34-h incubation under an Ar:H_2_:CO_2_:C_2_H_2_ atmosphere at 65°C (Table 1). Ethylene production was similarly detected in both strains.

In the present study, we isolated a *Thermodesulfovibrio* strain from Nakabusa Hot Springs that grew in the NH_3_-free modified JCM479 medium under an N_2_:CO_2_:H_2_ atmosphere. The genomic ana­lysis confirmed the presence of genes associated with the Calvin-Benson-Bassham cycle (Table S1) and N_2_ fixation (Fig. 1), and acetylene reduction assays demonstrated N_2_-fixing activity (Table 1). Bacteria in the genus *Thermodesulfovibrio* are well characterized as sulfate-reducing thermophiles (Maki, 2015); however, their N_2_ fixation ability has not been reported. CO_2_ fixation and the gene set for the Calvin-Benson-Bassham cycle were recently reported in a newly isolated strain, *T. autotrophicus*, from Stolbovsky hot spring, Russia (Maltseva *et al.*, 2024). However, this strain lacks N_2_-fixation-related genes. Our new isolate, strain TK110, is the first *Thermodesulfovibrio* strain to possess both N_2_- and CO_2_-fixing abilities.

The *nif* gene operon in strain TK110 consists of *nifHDKENXIIB*, similar to some *Thermodesulfovibrio* species, such as *T. yellowstonii*. Interestingly, strain TK110 exhibited the fusion of the *nifE* and *nifN* genes into a single open reading frame, whereas most diazotrophs possessed these genes separately (Nichio *et al.*, 2025). NifE and NifN comprise a multi-subunit enzyme, which is essential for nitrogenase maturation (Burén *et al.*, 2020). While the fusion of NifEN is uncommon (Nichio *et al.*, 2025), it has been shown to function in some cyanobacterial species (Thiel *et al.*, 1995). Downstream of *nifHDKENXIIB*, strain TK110 harbors a *nifA* homolog (TdN_17780), similar to other diazotrophic *Thermodesulfovibrio* species. NifA is a transcriptional activator for *nif* genes, including *nifH*, and is widely distributed in aerobic diazotrophs (Zhang *et al.*, 2023). Additionally, strain TK110 possessed NifI, a signal transduction protein commonly found in anaerobic diazotrophs, suggesting that NifA and NifI work together to regulate nitrogenase expression in response to nitrogen availability in *Thermodesulfovibrio* (Zeng *et al.*, 2024).

*Thermodesulfovibrio* species have been found in various thermal environments, including geothermal springs, thermal vent water in a lake, and anaerobic digesters (Maltseva *et al.*, 2024). Some strains exhibit CO_2_- and/or N_2_-fixing abilities and may contribute to carbon and nitrogen inputs into thermal environments. As previously reported, some species reduce nitrate, arsenate, or sulfite in addition to sulfate (Maltseva *et al.*, 2024). The high physiological diversity of *Thermodesulfovibrio*, including autotrophy and diazotrophy, highlights its ecological significance and makes its evolutionary traits a subject of interest for future research.

The 16S rRNA gene sequence and genomic sequence of strain TK110 were deposited in the DDBJ/EMBL/GenBank databases with the accession numbers LC874597 and BAAHNB010000001–BAAHNB010000002, respectively. The BioSample and BioProject accession numbers are SAMD00879867 and PRJDB20001, respectively. Raw sequence reads are available in the DDBJ Sequence Read Archive under the accession number DRR635008.

## Citation

Hisano, T., Nishihara, A., and Haruta, S. (2025) Nitrogen-fixing Ability of a Thermophilic Sulfate-reducing Bacterium in the Genus *Thermodesulfovibrio* Isolated from a Terrestrial Hot Spring in Japan. *Microbes Environ ***40**: ME25030.

https://doi.org/10.1264/jsme2.ME25030

## Supplementary Material

Supplementary Material

## Figures and Tables

**Fig. 1.**
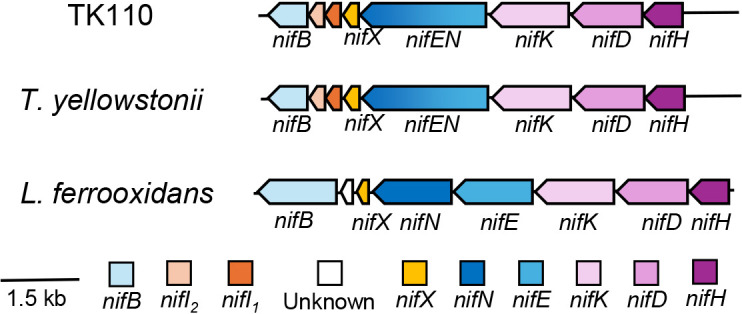
Nitrogen fixation gene clusters for strain TK110, *Thermodesulfovibrio yellowstonii* DSM 11347^T^, and *Leptospirillum ferrooxidans* C2-3^T^. Arrows indicate the transcriptional direction.

**Table 1.** Acetylene reduction activities of strain TK110 and its relative, *Thermodesulfovibrio yellowstonii* DSM 11347^T^.

	Cell density (cells mL^–1^)*	nmol C_2_H_4_ 10^4^ cell^–1^**
Strain TK110	(0.82±0.30)×10^6^	1.74±0.81
*T. yellowstonii*	(1.65±0.63)×10^6^	1.38±0.74

* Strain TK110 was pre-cultivated in the NH_3_-free modified JCM479 medium under an N_2_:CO_2_:H_2_ atmosphere, whereas *T. yellowstonii* DSM 11347^T^ was pre-cultivated in the NH_3_-free medium supplemented with lactate. Cell density at the growing phase was assessed after 5 days of pre-cultivation prior to the acetylene reduction assay. Values were obtained from three vials and are shown with standard deviations. The initial cell densities of these pre-cultures were <1×10^4^‍ ‍cells‍ ‍mL^–1^. ** The gas phase of the pre-cultures was replaced with Ar:CO_2_:H_2_ and supplemented with acetylene gas. Ethylene production was measured after 34‍ ‍h of incubation at 65°C. Values were obtained from three vials and are shown with standard deviations. Ethylene production from the autoclaved cells incubated under identical conditions was <0.05‍ ‍nmol 10^4^ cell^–1^.
