# Supplementary material for: Nitrogen-fixing Ability of a Thermophilic Sulfate-reducing Bacterium in the Genus Thermodesulfovibrio Isolated from a Terrestrial Hot Spring in Japan
Source: Microbes Environ. 2025 Sep 18;40(3):ME25030. doi: 10.1264/jsme2.ME25030 (PMC12501866; doi:10.1264/jsme2.ME25030)
Supplement: Supplementary file 1 — Supplementary Material [file 40_25030_s1.pdf]

Supplemental Materials

Title

Nitrogen-fixing ability of a thermophilic sulfate-reducing bacterium in the genus *Thermodesulfovibrio* isolated from a terrestrial hot spring in Japan

Authors

Toko Hisano,[1,2] Arisa Nishihara, [1,3], and Shin Haruta[1]

Affiliations

[1] Department of Biological Sciences, Tokyo Metropolitan University, 1-1 Minami-Osawa, Hachioji, Tokyo 192-0397, Japan

[2] Department of Biological Sciences, Purdue University, West Lafayette, IN 47907, USA

[3] Bioproduction Research Institute, The National Institute of Advanced Industrial Science and Technology (AIST), 1-1-1 Higashi, Tsukuba, Ibaraki 305-8566, Japan

Correspondence

Shin Haruta, sharuta@tmu.ac.jp

Supplemental Figure S1 Phase-contrast microscopic image of strain TK110.

The image was captured with optical microscope (Axio Imager A2, Carl Zeiss) equipped with a DP73 camera (Olympus) and CellSens standard software (Olympus). Bar, 10 μm.

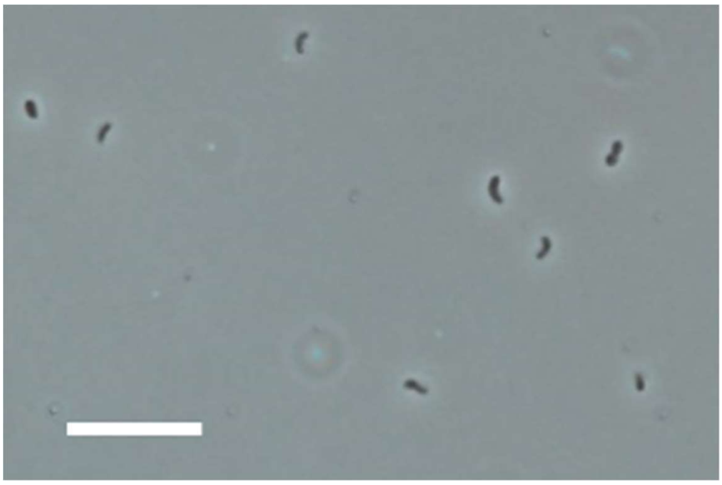

Supplemental Figure S2 Phylogenetic position of strain TK110 in the phylum *Nitrospirota*. The 16S rRNA gene sequences obtained and reference sequences were aligned using the Muscle program. The phylogenetic tree was constructed by the Maximum Likelihood method. The 16S rRNA gene sequences of *Chloroflexus aurantiacus* J-10-fl^{T} was added as an outgroup. Bootstrap values based on 100 resamplings are given at the nodes. Scale represents nucleotide substitutions per site.

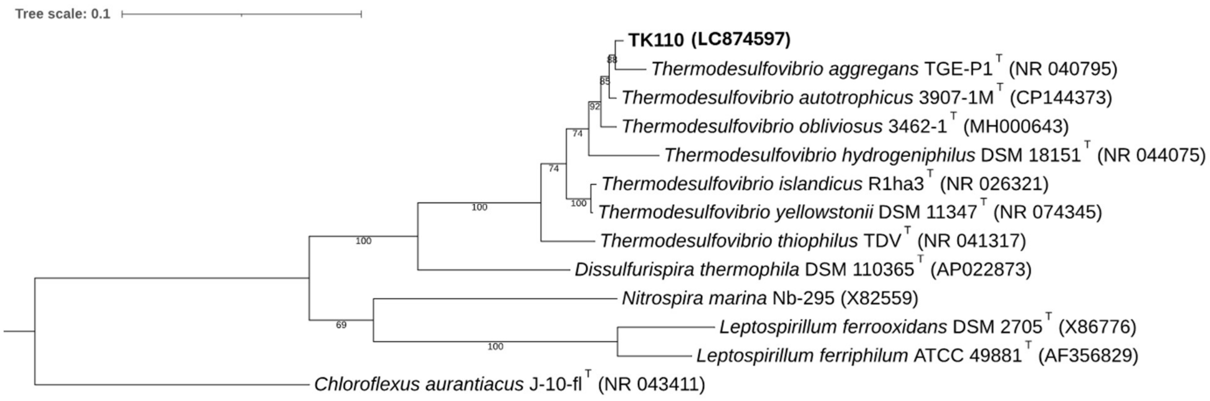

Supplemental Table S1 List of genes related to form III RubisCO-mediated transaldolase variant of the Calvin-Benson-Bassham cycle in the genome of strain TK110.

	Locus tag	Similarity*
Ribulose-phosphate 3-epimerase	TdN_07040	92.7
Thioredoxin reductase / NAD(P)-binding domain-containing protein	TdN_07030	89.4
Pyruvate kinase	TdN_07020	95.6
Class I fructose-bisphosphatase	TdN_07010	98.8
Class II fructose-bisphosphate aldolase	TdN_07000	96.3
Ribose-5-phosphate isomerase B	TdN_06970	97.9
Phosphoglycerate kinase	TdN_06980	97.5
NAD-dependent glyceraldehyde-3-phosphate dehydrogenase	TdN_06970	97.9
Transketolase, C-terminal section	TdN_06960	97.4
Transketolase, N-terminal section	TdN_06950	98.9
Phosphoribulokinase	TdN_06940	97.1
Ribulose-1,5-bisphosphate carboxylase, Type III	TdN_06930	98.4
Transaldolase	TdN_06920	98.6
Triosephosphate isomerase	TdN_06050	95.3
Class II fructose-bisphosphate aldolase	TdN_00990	97.5
NAD-dependent glyceraldehyde-3-phosphate dehydrogenase	TdN_14860	99.1
NAD-dependent glyceraldehyde-3-phosphate dehydrogenase	TdN_12310	99.5
Phosphoglycerate kinase	TdN_14870	99.5
Pyruvate kinase	TdN_11370	96.8
Class II fructose-bisphosphatase	TdN_12520	99.7

* similarity % with the deduced amino acid sequence from *Thermodesulfovibrio autotrophicus* (CP144373)
